# Exclusive breastfeeding among infants in Jigawa State, Nigeria: a cross-sectional analysis

**DOI:** 10.1186/s13006-025-00784-8

**Published:** 2025-11-24

**Authors:** Funmilayo Shittu, Adegoke G. Falade, Ayobami A. Bakare, Damola Bakare, Julius Salako, Susanne Rautiainen, Tim Colbourn, Rochelle A. Burgess, Carina King

**Affiliations:** 1 Department of Paediatrics, University of Ibadan, Ibadan, Nigeria; 2 Department of Global Public Health, Karolinska Institutet, Stockholm, Sweden; 3 Department of Community Medicine, University of Ibadan, Ibadan, Nigeria; 4 Clinical Epidemiology Division, Department of Medicine, Solna, Karolinska Institutet, Stockholm, Sweden; 5 Institute for Global Health, University College London, London, UK

**Keywords:** Infant, Exclusive breastfeeding, Complementary feeding, Nigeria

## Abstract

**Background:**

Initiation of breastfeeding within 1 hour of birth, exclusive breastfeeding (EBF) up to 6-months, and complementary feeding with continued breastfeeding up to two years of age are World Health Organization recommended practices. However, infant feeding patterns vary widely, influenced by cultural, social, economic and environmental factors. This study aims to evaluate EBF in Jigawa state, proving context-specific data to guide locally relevant public health interventions.

**Methods:**

We conducted a cross-sectional analysis of household survey data collected between January to June 2021 in Kiyawa Local Government Area, Jigawa. These data formed the baseline survey from the ‘Integrated Sustainable Childhood Pneumonia and Infectious Disease Reduction in Nigeria’ (INSPIRING) Jigawa cluster randomized controlled trial (ISRCTN3921355). We analysed a sub-set of the data, for women with children under two years of age, to determine the prevalence of early initiation of breastfeeding, EBF up to 6-months, and timing of complimentary feeding initiation, and associations with socio-economic indicators. Multivariable modified Poisson regression was used to explore associations with EBF amongst 0–5-month-olds (i.e. current EBF), and with retrospectively reported EBF up to 6 months amongst 6–24-month-olds.

**Results:**

A total of 4836 eligible women with a child under two years were surveyed across 3800 compounds. Among mothers of children aged 0–5 months, 98.1% were breastfeeding and 71.3% reported EBF at the time of the survey. Only 20.0% initiated breastfeeding within 1 hour of birth. The likelihood of EBF was lower among women in manual (aRR: 0.85; 95%CI: 0.77, 0.94) and non-manual work (aRR: 0.87; 95%CI: 0.78, 0.97) compared to those not working, and higher in those from higher compound wealth quintiles, and who had suffered recent pregnancy or child loss (aRR: 1.25; 95%CI: 1.01, 1.56). Among 6–24-month-olds, older maternal age reduced the likelihood of completing 6-months of EBF.

**Conclusion:**

EBF was associated with socio-economic factors in this rural, low-income setting, but the self-reported prevalence of EBF was much higher than expected, compared to nationally representative surveys. Targeted support for older mothers, mothers of twins, and household-centered education involving key family members will be essential to improve EBF practices in this high mortality setting.

## Background

Adequate nutrition is essential for the optimal growth and development of infants. The World Health Organization (WHO) and the United Nations Children’s Fund (UNICEF) recognize breastfeeding as the single most effective and affordable feeding practice for ensuring healthy development [1]. Consequently, they recommend early initiation of breastfeeding within one hour of birth, feeding infants with colostrum, exclusive breastfeeding (EBF) for the first six months of life, and continued breastfeeding up to two years of age [1]. These act as key indicators used for tracking infant and child health globally [2], given the role of early feeding practices in protecting newborns from infections and reducing infant mortality [3].

Data from Demographic Health Surveys (DHS) from 2002 to 2015 in 46 low- and middle-income countries (LMIC) reported early initiation of breastfeeding practices, and of these, 54% of countries had below 50% coverage - the global nutrition target set within the Sustainable Development Goals [4]. According to the 2023 Global Breastfeeding Scorecard report, despite progress being made, none of the global targets for breastfeeding have been met; 46% of newborns started breastfeeding within one hour of birth and 48% are exclusively breastfed for 6 months, falling far below the 2030 target of 70% set by WHO and UNICEF [5]. A range of newborn and infant feeding practices are seen worldwide [6, 7], with feeding patterns outside of EBF in the first six-months of life including breastfeeding supplemented with formula, formula feeding only, early initiation of complementary feeding, and formula plus complementary feeding [8–10]. Studies have shown that a mother’s breastfeeding practice is often influenced by several factors, which vary across contexts [11–13], including cultural, social, economic and even environmental factors [14–17].

From six months of age, breastmilk no longer provides infants with sufficient nutritional requirements to meet their energy needs, including key nutrients like iron, vitamin D and protein, and therefore complementary feeding is recommended [18]. Complementary feeding is the process of providing an infant with other foods and liquids to meet their nutrient requirements, and this is therefore a vulnerable period when malnutrition can set in [19]. Hence the WHO also recommends timely initiation of safe, nutritionally-adequate and age-appropriate complementary feeding at six months with continued breastfeeding up to two years of age [19].

In Nigeria, while breastfeeding is nearly universal with 98% of all children breastfed for a period of time, only 31% of newborns are breastfed within the first hour of birth, and just 29% of infants under six-months are exclusively breastfed – far below the global average, and this figure has stagnated from 2018 to 2024 [20]. Malnutrition is also common in Nigeria, with approximately 2 million children suffering from severe acute malnutrition in 2015 and is ranked second in the world for the burden of stunting – a low height-for-age [21]. The 2024 DHS reported no improvements in stunting, wasting and underweight among under-5 children with these indicators actually worsening since the 2018 DHS assessment [20]. This high prevalence of malnutrition can be linked to poor feeding practices, with the low adoption of EBF and sub-optimal introduction of complementary feeding. Sub-national inequities are also present, with Jigawa state, in the North West, having only 15.6% of infants under six months exclusively breastfed compared, and just 8% reporting initiation of breastfeeding within the first hour.

We therefore aimed to evaluate the coverage of EBF among women with children under-two years in Jigawa state, northern Nigeria, in order to generate context-specific evidence that can inform targeted public health strategies to improve infant nutrition and survival in this high-mortality, underserved population.

## Methods

### Study design

We conducted a cross-sectional analysis of household survey data collected between January to June 2021 in Kiyawa Local Government Area (LGA), Jigawa State, Nigeria. These data formed the baseline survey from the INSPIRING Jigawa cluster randomized controlled trial (ISRCTN3921355, registered: 11^th^ December 2019). [22, 23]. We analysed a sub-set of the data, for women with children under two years of age, to determine the prevalence of early initiation of breastfeeding, EBF up to 6-months (current EBF in children < 6 months and completed EBF in children 6–24 months), and associations with socio-economic indicators. Full methods for sampling and data collection have been published previously [23].

### Setting

Jigawa state has an estimated population of 4.3 million constituting mainly Hausa-Fulani tribes, and a small proportion of Manga and Badawa, tribes [24]. This study was done in Kiyawa LGA which consists of 3 districts: Kiyawa district, Shuwarin district and Abalago district, with a total of 11 wards - administrative units used for electoral purposes. People live in extended family compounds typically comprising of two to five households with extended families of children, parents, grandparents and other siblings sharing resources. The setting is culturally homogenous, with 99% of the population practising Islam, and 91% of the people engaged in agricultural practices as their main occupation. Two-thirds of the population (69%) live in severe poverty, with 50% belonging to Nigeria’s lowest wealth quintile [24].

Child mortality in Jigawa was 161/1,000 livebirths according to the 2024 DHS, and malnutrition is a major challenge. Recent surveys estimate that around 6–8% of under 5 children suffer from severe acute malnutrition, and a further 15–20% experience moderate acute malnutrition [25]. In addition, the prevalence of stunting affects approximately 68% of children under five, among the highest rates in Nigeria. Antenatal care seeking was 37.7% of women attending more than 4 ANC visits, compared to the national average of 52% [26].

### Study population

The study population was women with children under two years of age at the time of the survey, residing in selected compounds in villages within Kiyawa LGA who were considered permanent residents.

### Sampling

We conducted an LGA-wide mapping of compounds in 2020 to form the sampling frame. We then used simple random sampling proportional to the INSPIRING trial cluster size, to select a minimum of 50 compounds per cluster across the 32 trial clusters using a random number generated in Stata version SE14. In selected compounds, all women aged 16–49 years who were permanent residents were eligible for inclusion.

### Data collection

We recruited and trained 36 data collectors who underwent one week of training, with 3 days of in-class and 3 days field training. The study data collectors asked eligible women questions about the time they initiated breastfeeding (for children aged 0–3 months only), EBF to 6-months, and initiation of complimentary feeding for each of their surviving children. Data was collected using Android tablets, with a custom project CommCare application, which linked compound, woman and child data collection forms.

### Analysis

Descriptive statistics were calculated by using frequencies and percentages for categorical variables and means and standard deviation for continuous variables. Cross tabulation and chi-square tests were used to show univariate associations. The primary analysis was done amongst children aged 0–5 months to estimate the association between ‘current EBF’ and sociodemographic factors using multivariable-adjusted modified poisson regression, to produce relative risks [27]. A secondary analysis was done, using the same approach, amongst children aged 6–24 months. EBF was defined based on women’s self-reported responses to whether they were currently breastfeeding, had given the child any other forms of liquids or foods, and if yes, how old was the child in months when this was introduced. This was adapted from the DHS approach, although we did not explicitly ask about and include oral rehydration solution, vitamins, mineral supplements or medicines in the definition.

Sociodemographic variables of interest included: child sex, type of pregnancy, woman’s education, woman’s age, woman’s first marriage, wife ranking, age at first marriage, woman’s occupation, compound wealth. A theory based approach was used to choose the independent variables in the analysis, by considering factors which have been previously reported as relevant in this context [16, 28]. We also included having experienced a pregnancy or child loss in the previous 12 months as a variable of interest, as we have previously observed an association with pneumonia care-seeking for surviving children in this setting [29]. Principal components analysis was used to create the compound wealth quintile, based on asset ownership and access to water, toilet facilities and power.

We also explored the potential role of education as an effect modifier. A complete case analysis approach was used, as participants with incomplete questionnaire responses were uncommon. Data cleaning was conducted to remove illogical responses. Variables with a P-value < 0.250 in univariate analyses were retained for the adjusted multivariate analyses. All analysis was conducted using Stata SE version 14.

## Results

### Study participants

**Fig. 1:**
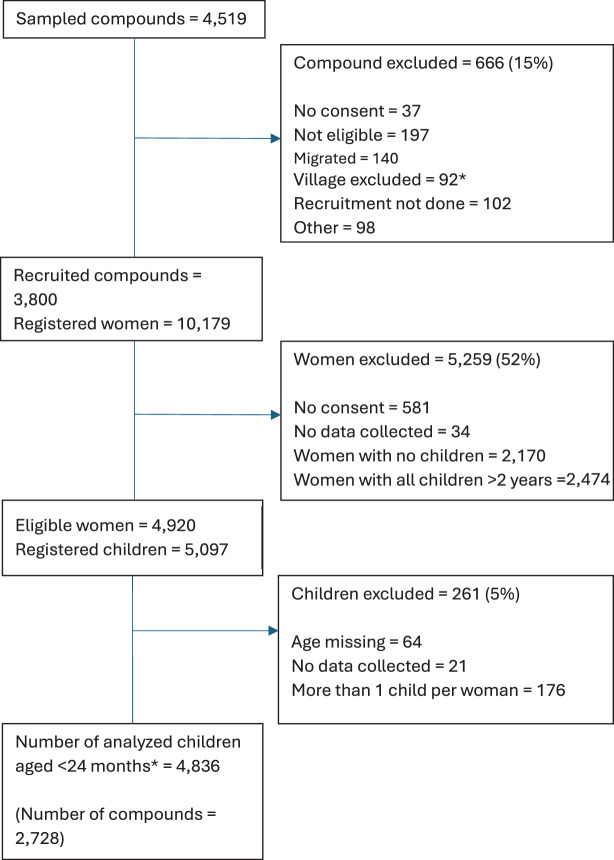
Consort diagram for women’s inclusion *If a woman had more than one child aged 0–24 months, the youngest child was selected; in the case of multiple births (i.e. twins and triplets), one child was randomly selected

Overall, there were 4836 eligible women with a child under-two years, out of the 10,179 women recruited from 3800 compounds in the survey – Fig. 1. The mean compound size was 23 people (SD: 18.9), and a mean of 1.78 eligible women per compound included for this study (range 1–13). More than two-thirds (*n* = 1265) of mothers were aged 21–25, 50.5% (*n* = 2444) had 2 children, 62.5% (*n* = 3024) were engaged in manual labour and only 7.2% (*n* = 382) had any formal education. In terms of marriage, the vast majority of women were married (98.6%), with most in their first marriage (81.9%), and 90.9% got married before the age of 18 years old (Table 1). Of the 4836 children, 27% (*n* = 1310) were aged 0–5 months, 49% (*n* = 2411) were male and 97.4% were born as singletons (Table 2).

**Table 1:** Women’s and compound characteristics (*N* = 4,836)

Socio-demographic characteristics		n	%
Compound variables			
Number of people living in a compound	1–20	2751	56.9
	21–50	1718	35.5
	> 50	367	7.6
Head of compound gender	Female	125	2.6
	Male	4711	97.4
Head of compound age	20–39	478	9.9
	40–59	2151	44.5
	60–79	1726	35.7
	≥80	461	9.5
	Missing	20	0.4
Head of compound highest level of education	No formal education	982	20.3
	Informal/religious education	3020	62.5
	Primary	353	7.3
	Secondary	273	5.7
	Tertiary/further	203	4.2
	Missing	5	1.1
Head of compound marital status	Currently married	4706	97.3
	Not currently married	130	2.7
Head of compound main occupation	Farming	2441	50.5
	Manual labour	595	12.3
	Business owners	1404	29.0
	Professional/TBA	204	4.2
	Not working	178	3.7
	Missing	14	0.3
Wealth status*	Lowest	776	16.1
	Low/middle	964	19.9
	Middle	955	19.8
	Middle/high	991	20.5
	Highest	1150	23.8
Woman characteristic			
Age	16–20	1104	22.8
	21–25	1265	26.2
	26–30	1141	23.6
	31–35	654	13.5
	36–40	426	8.8
	41–49	244	5.1
Occupation**	No formal work	462	9.6
	Manual labour	3024	62.5
	Non-manual labour	1350	27.9
Highest level of education	No formal education	1806	37.3
	Informal/religious education	2644	54.7
	Formal education	382	7.9
	Don’t know	4	0.1
Number of children under-five	1	2184	45.2
	2	2444	50.5
	3	193	4.0
	4	15	0.3
Marital status	Married – first marriage	3958	81.9
	Married – second marriage	809	16.7
	Not currently married	69	1.4
Age at first marriage	10–14	1070	22.1
	15–16	3322	68.7
	> 17	441	9.1
	Missing	3	0.1
Pregnancy or child loss in the prior 12 months	No	4801	99.3
	Yes	35	0.7

*Compound wealth was calculated using a principal component analysis of shared compound assests and access to water, toilet facilities and power

**No formal work included: housework, retirement, student; manual labour included: subsistence farming, unskilled and skilled manual labour (e.g. cap knitting); non-manual labour included: commercial farming, small business owner, professional role, TBA

**Table 2:** Child characteristics and feeding behaviours (*N* = 4836)

		Overall		Children aged 0–5 months (*n* = 1310)		Children aged 6–24 months (*n* = 3526)	
		n	%	n	%	n	%
Sex	Male	2411	49.2	669	50.8	1742	49.6
	Female	2378	49.9	626	48.1	1752	49.6
	Missing	47	1.0	15	1.2	32	0.9
Type of pregnancy	Singleton	4709	97.4	1273	97.2	3437	97.4
	Twin	80	1.7	22	1.7	57	1.7
	Missing	47	1.0	15	1.2	32	0.9
Time of breastfeeding initiation* (*N* = 872)	< 1 hour	173	20.0				
	1–6 hours	321	36.7				
	6–12 hours	191	21.9				
	12–24 hours	143	16.4				
	> 24 hours	38	4.4				
	Missing	6	0.7				
Currently breastfeeding	Yes	3988	82.5	1285	98.1	2703	76.7
	No	848	17.5	25	1.9	823	23.3
Currently complementary feeding	Yes	3648	75.4	361	27.6	3287	93.2
	No	1188	24.6	949	72.4	239	6.8
Month of complementary feeding initiation (mean, SD)		5.22	2.47	2.07	1.79	5.49	2.33
Month of breastfeeding termination (mean, SD)		18.49	4.65	2.22	1.76	19.95	3.81
Exclusively breastfed**	Not exclusive	1545	32.0	369	28.2	1176	33.4
	Exclusive	3291	68.0	941	71.3	2350	66.7

*Only asked to children 0–3 months

**For children aged 6–24 months, this represents completed EBF to 6 months of age

### Breastfeeding and complimentary feeding prevalence

Overall, 98.1% of women reported currently breastfeeding their children aged 0–5 months, but 27.5% had also started complimentary feeding, with 71.3% of children aged 0–5 months being exclusively breastfed (Table 2). In this age group, for the sub-group of women who stopped breastfeeding before the child reached 6 months of age, the mean month of termination was 2.2 months, similar to the mean month when they reported starting complementary feeding (mean: 2.1 months). In terms of initiation of breastfeeding, only 20.0% of women with a child aged 0–3 months reported initiating breastfeeding within 1 hour of birth, with 94.9% starting breastfeeding in the first 24 hours.

**Fig. 2:**
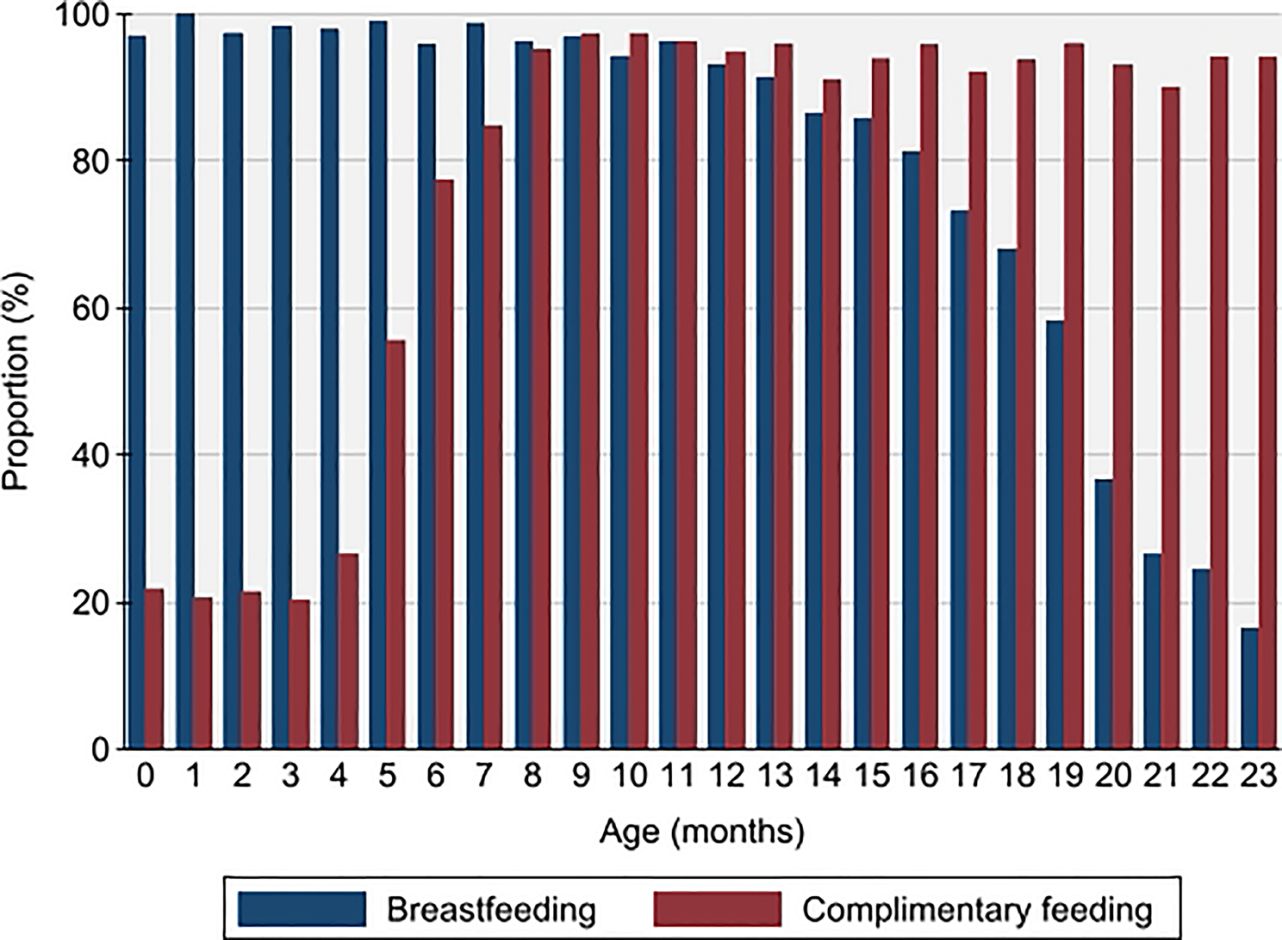
Proportion of children breastfeeding and complimentary feeding, by age in months

For children aged 6–24 months, 76.7% of mother reported that they were still breastfeeding, and 93.2% had started complimentary feeding, with 66.7% self-reporting that they completed 6 months of EBF (Table 2). Overall, women breastfed for a mean of 18.5 months and introduced complimentary feeding at 5.2 months on average (Fig. 2).

### Socio-economic associations with current EBF

In the bivariate analysis, women’s occupation and compound wealth were significantly associated with current EBF among children aged 0–5 months and were retained alongside education and pregnancy or child loss in the adjusted model. The likelihood of EBF was lower among women engaged in manual labour (aRR = 0.85; 95% CI: 0.77, 0.94) and non-manual labour (aRR: 0.87, 95% CI: 0.78, 0.97) compared to women without formal work. For compound wealth, there was an increasing trend of current EBF with higher wealth, with women in the highest wealth quintile having 26% higher likelihood (aRR: 1.26, 95% CI: 1.11, 1.42) of current EBF than those in the lowest quintile (Table 3). Women who experienced a pregnancy or child loss in the prior 12-months were 25% (aRR: 1.25, 95%CI: 1.01, 1.56) more likely to be currently practicing EBF than those who had not. Child sex, type of pregnancy, woman’s age, education and marital status were not associated with current EBF.

**Table 3:** Association between socio-economic factors and self-reported exclusive breasting practices for children aged 0–5 months

		Exclusively breastfed		Crude RR	95% CI	p-value	Adjusted RR	95% CI	p-value
		n/N	%						
Child Sex	Female	455/629	72.3	1.00					
	Male	480/666	72.1	0.99	0.93, 1.07	0.936			
Type of pregnancy	Singleton	919/1273	72.2	1.00					
	Multiple	16/22	72.7	1.01	0.78, 1.30	0.962			
Woman’s education	No formal	330/472	69.9	1.00			1.00		
	Informal	518/718	72.1	1.03	0.96, 1.11	0.455	1.03	0.96, 1.11	0.430
	Any formal	92/119	77.3	1.10	0.98, 1.24	0.093	1.04	0.93, 1.17	0.510
Woman’s age	16–20	243/338	71.9	1.00					
	21–25	244/332	73.5	1.03	0.94, 1.13	0.580			
	26–30	225/314	71.7	1.00	0.91, 1.10	0.946			
	31–35	114/164	69.5	0.97	0.86, 1.09	0.586			
	36–40	70/101	69.3	0.96	0.83, 1.12	0.623			
	41–49	44/60	73.3	1.02	0.86, 1.20	0.816			
Marital status	First marriage	789/1091	72.3	1.00					
	Second marriage	147/211	69.7	0.96	0.87, 1.06	0.432			
	Unmarried	5/8	62.5	0.86	0.50, 1.48	0.592			
Woman’s Occupation	No formal work	106/132	80.3	1.00			1.00		
	Manual labour	578/825	70.1	0.87	0.79, 0.96	0.006	0.85	0.77, 0.94	0.001
	Non-manual labour	257/353	72.8	0.91	0.82, 1.01	0.070	0.87	0.78, 0.97	0.013
Compound wealth	Lowest	135/217	62.2	1.00			1.00		
	Low/middle	181/265	68.3	1.10	0.96, 1.25	0.166	1.11	0.97, 1.27	0.123
	Middle	179/248	72.2	1.16	1.02, 1.32	0.024	1.17	1.03, 1.33	0.020
	Middle/high	201/265	75.9	1.23	1.08, 1.39	0.001	1.24	1.09, 1.40	0.001
	Highest	245/315	77.8	1.25	1.11, 1.41	< 0.001	1.26	1.11, 1.42	< 0.001
Pregnancy or child loss in the prior 12 months	No	932/1300	71.7	1.00			1.00		
	Yes	9/10	90.0	1.25	1.02, 1.55	0.034	1.25	1.01, 1.56	0.043

### Socio-economic associations with completed ebf up to 6-months

For the analysis amongst the 6–24-month-olds, the child’s sex, woman’s marital status, occupation and having a child or pregnancy loss in the prior 12-months were not associated with having completed 6 months of EBF (Table 4). Increasing maternal age was associated with a lower likelihood of completed EBF, with 54.4% women aged 41–49 years compared to 73.8% of women aged 16–20 years reporting EBF up to 6 months (aRR: 0.75, 95% CI: 0.65, 0.85). Increasing compound wealth and having any formal education were associated with increased completed EBF, while the risk was 18% lower for children that were born as twins (aRR: 0.82, 95% CI: 0.65, 1.02).

**Table 4:** Association between socio-economic factors and completing ebf for 6-months, amongst children aged 6–24 months

		Exclusively breastfed		Crude RR	95% CI	p-value	Adjusted RR	95% CI	p-value
		N	%						
Child Sex	Female	1152/1748	65.9	1.00					
	Male	1176/1746	67.4	1.02	0.97, 1.07	0.409			
Type of pregnancy	Singleton	2296/3436	66.8	1.00			1.00		
	Multiple	32/58	55.2	0.84	0.67, 1.05	0.125	0.82	0.65, 1.02	0.070
Woman’s education	No formal	860/1334	64.5	1.00			1.00		
	Informal	1290/1926	67.0	1.04	0.99, 1.09	0.132	1.03	0.97, 1.08	0.330
	Any formal	200/263	76.1	1.18	1.09, 1.28	< 0.001	1.09	1.01, 1.18	0.031
Woman’s age	16–20	565/766	73.8	1.00			1.00		
	21–25	620/933	66.5	0.90	0.85, 0.96	0.001	0.90	0.84, 0.95	< 0.001
	26–30	544/827	65.8	0.89	0.84, 0.95	0.001	0.89	0.83, 0.95	< 0.001
	31–35	318/490	64.9	0.88	0.81, 0.95	0.001	0.88	0.81, 0.95	0.001
	36–40	202/325	62.2	0.84	0.77, 0.93	< 0.001	0.84	0.76, 0.92	< 0.001
	41–49	100/184	54.4	0.74	0.64, 0.85	< 0.001	0.75	0.65, 0.85	< 0.001
Woman’s first marriage	First marriage	1914/2867	66.8	1.00					
	Second marriage	394/598	65.9	0.99	0.93, 1.05	0.671			
	Unmarried	42/61	68.9	1.03	0.87, 1.22	0.728			
Woman’s Occupation	No formal work	216/330	65.5	1.00					
	Manual labour	1468/2199	66.8	1.02	0.94, 1.11	0.633			
	Non-manual labour	666/997	66.8	1.02	0.93,1.12	0.657			
Compound wealth	Lowest	320/559	57.3	1.00			1.00		
	Low/middle	441/699	63.1	1.10	1.01, 1.21	0.037	1.09	1.00, 1.19	0.062
	Middle	454/707	64.2	1.12	1.02, 1.23	0.013	1.10	1.01, 1.21	0.032
	Middle/high	500/726	68.9	1.20	1.10, 1.31	< 0.001	1.19	1.09, 1.29	< 0.001
	Highest	635/835	76.1	1.33	1.23, 1.44	< 0.001	1.31	1.21, 1.43	< 0.001
Pregnancy or child loss in the prior 12 months	No	2333/3501	66.6	1.00					
	Yes	17/25	68.0	1.02	0.78, 1.34	0.886			

## Discussion

In this study, we explored the association of different socio-economic factors with EBF among children aged 0–5 months olds (i.e. currently practising EBF), and aged 6–24 months old (i.e. completed the recommended 6-months of EBF), in a rural setting in Jigawa State, Northern Nigeria. Overall, breastfeeding was nearly universal (98.1%), and the prevalence of current EBF was higher than expected, at 71.3% amongst children aged 0–5 months. Higher compound wealth was associated with both current and completed EBF, but other socio-demographic factors were not consistently associated with both outcomes, suggesting education, occupation, pregnancy/child loss and type of pregnancy influence practices in different ways.

The predominately breastfeeding rate of 71.3% among children aged 0–5 months is considerably higher than the 2024 Nigeria DHS value of 28.8% and 2021 MICs value of 34.4% [30]. However, other infant feeding indicators were closer, with the MICs reporting 88% of children in Jigawa State being breastfed, 7.8% initiating feeding within 1 hour of birth, and an average of 20.7 months of breastfeeding. Interestingly, they report that 80.4% of children in Jigawa are “predominantly” breastfed, defining this as “*infants who receive breast milk and certain fluids (water and water-based drinks, fruit juice, ritual fluids, oral rehydration solution, drops, vitamins, minerals, and medicines), but do not receive anything else*” [30]. It is therefore likely, that our data on EBF actually reflects ‘predominantly EBF’, where women did not see water, or other fluids and medications, as a violation of EBF. Our previous qualitative work would support this, as water (and water mixed with other liquids), had several symbolic and traditional roles during infant feeding [16], and was commonly reported as being part of EBF (ref: c1853862-3dad-4656-9f29-46e4620936b6). This community misunderstanding around water as part of EBF has also been reported from other contexts [31, 32]. Exploring the extent to which water, and other liquids, are accepted as part of EBF should be explored further to support reliable and comparable data across settings. This is also important for the development of context appropriate public health messaging that conveys the rationale behind the WHO recommendation [33].

Mothers with formal education exhibited higher odds of completing 6-months of EBF, but not current EBF, while maternal occupation showed the opposite patterns, with women engaged in either manual (subsistence farming, knitting, petty trading) or non-manual labour (TBAs, commercial farming, civil servants) having lower odds of current EBF. While these findings may also reflect social desirability bias due to the self-reported nature of feeding practices, previous studies from Nigeria and similar sub-Saharan African contexts have highlighted how maternal work poses significant challenges to EBF adherence due to competing demands on maternal time [34–36]. In Jigawa’s conservative social setting, women’s work is typically within the informal sector, and is home-based or near-home (e.g., petty trading, knitting, farming on nearby land). This means their children would generally be with them, but given the care of children in this context is often shared amongst other women and is intergenerational [37], others would step in while the woman is busy working. This finding may therefore reflect the critical role of intra-household power dynamics and social norms in shaping maternal and child health behaviors in Jigawa.

Grandmothers, mothers-in-law, and male heads of household exert significant influence over infant feeding decisions, often discouraging EBF in favour of early water initiation [16]. This aligns with findings from other African contexts, where intra-household hierarchies shape breastfeeding practices, underscoring the importance of engaging not just mothers but entire household networks in breastfeeding promotion [38, 39]. This generational shift in practices is also reflected in our finding that older women had lower odds of completing 6-months of EBF. This may reflect more traditional beliefs, but also having more household responsibility with multiple children or having an occupation – while younger women more often reporting not working meaning they had more opportunity to breastfeed. These factors are also linked to compound wealth, as women from compounds with more resources may be more able to take a break from their work to breastfeed, but also access post-partum support.

In this setting, early marriage is common, limiting women’s opportunities for formal education. Consequently, most mothers acquire knowledge of childcare and feeding through day-to-day practices and experiential learning passed down from older women, relatives, and co-wives within their households. This form of practical, culturally embedded education has a significant influence on breastfeeding practices in Jigawa, where social norms and informal mentorship systems shape health behaviors more profoundly than formal schooling. Similar patterns have been documented in other northern Nigerian and sub-Saharan African settings, where community-based, experience-driven knowledge systems play a key role in maternal and child health practices [9, 40]. While formal education remains an important factor, in settings characterized by early marriage and low female literacy rates, integrating culturally appropriate, community-based health education interventions are likely needed to promote EBF completion for the full 6-months. It is also important to consider the role of influential household members as advocates for change and therefore support their education on the benefits of EBF and the potential risks associated with early introduction of other liquids. Their support could empower mothers to adhere to EBF guidelines, even in the face of societal pressures, leading to broader cultural transformations as new norms are established and reinforced within the community.

While it was not a statistically significant finding, with small numbers, it was interesting to observe the association between recent pregnancy or child loss had higher odds of current EBF. It’s possible that women respond to the trauma of loss, by taking additional steps to protect their current pregnancy and newborn – but as the child survives, the additional fear of loss diminishes. This resonates with existing literature from other African settings, where personal experiences of child loss act as a potent motivator for adopting recommended health behaviors in subsequent pregnancies [41, 42]. In the sociocultural context of Jigawa, where infant and child mortality remain high—often exacerbated by preventable causes such as diarrhoea from unsafe water and early complementary feeding—such experiences of loss may serve as a trigger for behavioural change among affected mothers. Given the uncertainty of our findings, this is an area which needs further understanding to provide specific support for these mothers. Involving them as peer supporters or role models, could enhance EBF uptake and sustainability within communities facing high infant mortality rates.

Mothers of twins had lower odds of completing 6-months of EBF compared to mothers of singletons, although there was no difference in currently breastfeeding 0–5-month-olds. The physical demands of nursing two infants, coupled with competing household responsibilities and the absence of targeted support systems, likely make maintaining EBF particularly difficult for twins. Similar patterns have been reported in other African settings, where mothers of multiples experience early mixed feeding due to increased nutritional requirements, breastfeeding fatigue, and prevailing beliefs that breast milk alone is insufficient for twins [34, 43]. Importantly, current national and international breastfeeding policies and programs in Nigeria and elsewhere predominantly focus on mothers of singletons, often failing to capture the unique needs and vulnerabilities of mothers of multiple births [44]. Given twins have three times the mortality rate of singletons in Sub-Saharan Africa [45], designing specific interventions to support safe and supported infant feeding in northern Nigeria should be explored.

This study had two key limitations. Firstly, breastfeeding and complimentary feeding practices were self-reported by women, and therefore subject to recall, measurement and social desirability biases. As discussed above, it is likely that we had measurement bias in the reporting of EBF, with misunderstandings about the role of water as part of EBF [46]. Social desirability bias is also seen in self-reported EBF measures, and this study, for example if more educated women have more awareness of the benefits of EBF and therefore self-reported this practice more often, this could explain finding an association [47]. Self-reported data is the global norm for estimating EBF, and there are several methods which can be used to produce estimates of EBF. Our approach was in line with globally accepted norms, using a since birth recall for current EBF, and based on age of liquid/food introduction for completed EBF [48]. The biases associated with self-reported practices are acknowledged; however, they are unlikely to be substantially different from those observed in the wider literature on EBF estimates and associations. Secondly, as the cross-sectional survey was designed for the baseline of a cluster randomised controlled trial, it was not designed to be powered to address this research question specifically, or capture all possible variables associated with EBF practices (such as post-partum care). While the sample was large, and representative of Kiyawa LGA, some of the associations were likely underpowered, with small numbers of events – namely, having experienced a pregnancy or child loss.

Overall, this study found several socio-economic factors that were relevant to the practice of EBF within a rural, low-income context of Jigawa State, where child mortality and malnutrition are high. While EBF was reported to be high, this may reflect the cultural norm of water within infant feeding that was previously observed in this setting, and is an area that should be further explored develop context-appropriate feeding interventions. Culturally informed and household-centered interventions need to be developed, particularly to support mothers with twins and older mothers, who faced particular challenges in sustaining EBF for the recommended 6-months in this setting. Strengthening community education and involving influential family members will be critical to improving EBF uptake and reducing infant morbidity.

## Data Availability

Yes, data are available on request. Contact the following people with request; Carina King- carina.king@ki.se Colbourn Tim- t.colbourn@ucl.ac.uk Adegoke Falade- afalade33@hotmail.com.
